# Supplementary material for: A meta-analysis of pre-pregnancy maternal body mass index and placental DNA methylation identifies 27 CpG sites with implications for mother-child health
Source: Commun Biol. 2022 Nov 30;5:1313. doi: 10.1038/s42003-022-04267-y (PMC9709064; doi:10.1038/s42003-022-04267-y)
Supplement: Supplementary file 2 — Supplementary Information [file 42003_2022_4267_MOESM2_ESM.pdf]

Supplementary Note 1: Description of participating cohorts

AQUA (Asking QUestions about Alcohol in Pregnancy)

Design and study population

The AQUA (Asking QUestions about Alcohol) cohort was established to study the effects of different patterns of prenatal alcohol exposure in the offspring[1]. Recruitment was conducted between July 2011 to July 2012 in several metropolitan public hospitals in Victoria, Australia. Women aged 16 years and above, who were less than 19 weeks in gestation, with an uncomplicated singleton pregnancy, and who were sufficiently proficient in English were considered eligible for the study. Participants provided written informed consent and this study was approved by the Human Research Ethics Committees of Eastern Health (E54/1011), Mercy Health (R11/14), Monash Health (11071B), the Royal Women's Hospital (11/20), and the Royal Children's Hospital (31055A).

Placenta collection and DNA extraction

A total of 248 placentas were collected out of the total 1570 recruited participants. Three sections from each placenta were obtained randomly using a 6mm disposable biopsy punch, which were subsequently washed with sterile phosphate-buffered saline (PBS), dried, and then preserved in RNALater (Qiagen, Venlo, Netherlands) at 4˚C for 72 hours. The central segment of each biopsy was then dissected, and pooled in a single vial, followed by storage at -20˚C until DNA extraction. Genomic DNA (gDNA) from 117 placental samples were extracted using the phenol/chloroform followed by ethanol precipitation method as previously described[2]. NanoDrop spectrophotometer (Thermo Scientific, Waltham, MA, USA) was used to determine quality of gDNA.

Placental genome-wide DNAm data acquisition, quality control and normalization

A total of 500ng of the extracted gDNA samples were sent to Service XS (Leiden, The Netherlands) to generate genome-wide methylation data on the Illumina HumanMethylation450 (HM450) array. Out of the 117 samples, 7 (6 %) were excluded in this analysis because they either had missing data for maternal BMI (n=4) or had missing data for parity (n=3). Out of 110 samples, 95 were of white (Australian/UK/other European) ethnic origin. Data was normalised based on Beta-Mixture Quantile (BMIQ) normalisation method and batch effect of 10 arrays was adjusted using ComBat Package in R.

Genome-wide differential DNAm analyses

Estimation of cell type proportions was performed and estimated three cell types in our data. Robust linear regression models were used for genome-wide methylation analyses, adjusting for maternal age, parity, maternal education, maternal smoking during pregnancy, and maternal ethnicity.

Acknowledgements

The AQUA team members are very grateful to all the women and their children who are taking part in this study and wish to thank the researchers who were involved in sample collection, as well as Dr Sharon Lewis for providing the covariates for analysis.

Funding

This work is supported by the Australian National Health and Medical Research Council (Grant #1011070; Senior Research Fellowship #1021252 (JH)) and the Victorian State Government's Operational Infrastructure Support Program. Funding was also received from the McCusker Charitable Trust to assist with the biospecimen collection at birth.

EARLI (Early Autism Risk Longitudinal Investigation)

Design and study population

The Early Autism Risk Longitudinal Investigation (EARLI) is an enriched risk prospective pregnancy cohort to study autism etiology[3]. The EARLI study was reviewed and approved by Human Subjects Institutional Review Boards (IRBs) from each of the four study sites (Johns Hopkins University, Drexel University, University of California Davis, and Kaiser Permanente Northern California). This longitudinal study recruited mothers of confirmed ASD children who were early in a subsequent pregnancy or were trying to become pregnant. There were 232 mothers with a subsequent sibling born through this study. All siblings were born between November 2009 and March 2012. Demographics, maternal behaviors, food frequency, medical history were all collected via questionnaire. Biosamples and house samples were collected during pregnancy, at birth, and during development.

Placenta collection and DNA extraction

Placental biopsy samples were collected after delivery at each clinical lab site using Baby Tischler Punch Biopsy Forceps. Paired placental samples in EARLI were taken from the maternal and the child-facing sides of the placenta. Samples were stored at ambient temperature in RNAlater vials (Qiagen) and shipped same-day to the Johns Hopkins Biological Repository (JHBR) in Baltimore, Maryland, for storage at -190°C until DNA processing. Biospecimens including cord blood and placenta were collected and archived at 213 births. Placenta DNA was extracted using the DNA Midi kit (Qiagen, Valencia, CA) and samples were bisulfite treated and cleaned using the EZ DNA methylation gold kit (Zymo Research, Irvine, CA).

Placental genome-wide DNAm data acquisition, quality control and normalization

DNA was plated randomly and assayed on the Infinium HumanMethylation450 BeadChip (Illumina, San Diego, CA) at the Johns Hopkins SNP Center, a shared lab and informatics operation with the Center for Inherited Disease Research (Johns Hopkins University). DNA methylation control gradients and between-plate repeated tissue controls were used. There were 132 maternal-side placenta derived samples and 134 sibling-side placenta samples that passed DNA methylation quality control, 107 of which had complete data for covariates. For this analysis, only the sibling-side placenta DNA methylation measures were used. Beta-Mixture Quantile (BMIQ) normalization was done, followed by use of ComBat two adjust for batch effect of the two dates arrays were run.

Genome-wide differential DNAm analyses

Reference-free cell type deconvolution estimated three cell types, and two were used in models for adjustment. Robust linear regression models were performed for genome-wide methylation analyses. Covariates for adjustment include maternal age, maternal education (dichotomous having college degree or not), two ancestry PCs from genotype data, and cell type estimates. Parity was not included as a covariate, as all mothers in the study had a previous child by design. Only 6 mothers had any smoking during pregnancy and were excluded from analysis. 54 mothers in analysis set were of European ancestry.

Acknowledgements

We thank the participants of the EARLI study.

Funding

Funding for the EARLI study was provided by the National Institutes of Health (R01 ES016443, R24ES030893, R01ES025531) and Autism Speaks (003953). Mr. Dou and Dr. Bakulski were supported by grants from the National Institutes of Health (R01 ES025531, R01 MD013299). The content is solely

the responsibility of the authors and does not necessarily represent the official views of the National Institutes of Health.

EDEN (Study on the pre-and early postnatal determinants of child health and development)

Design and study population

The *Etude des Déterminants pré et post natals du développement et de la santé des Enfants* (EDEN) is a population-based mother-child cohort study in France that aim to study the role of pre- and post-natal factors in relation to child growth and development.[4] Women were recruited between 2003 and 2006. More information about the EDEN cohort is available through our webpage http://eden.vjf.inserm.fr/. The EDEN cohort received approval from the ethics committee (CCPPRB) of Kremlin Bicêtre and from the French data privacy institution "*Commission Nationale de l'Informatique et des Libertés*" (CNIL). Written consent was obtained from the mother for herself and for the offspring.

Placenta collection and DNA extraction

Overall, 1907 mother-child pairs were followed from until birth and placenta samples were collected for 1301 women. Placentas samples were collected at delivery by the midwife or the technician of the study using a standardized procedure. Samples of around 5mm x 5 mm were carried out in the centre of the placenta on the foetal side and were stored at −80 °C until processing. DNA from placental samples was extracted using the QIAsymphony instrument (Qiagen, Germany).

Placental genome-wide DNAmdata acquisition, quality control and normalization

For this particular study, genome-wide DNAm examination was performed in 664 placentas samples[5]. The DNAm analysis was performed by the *Centre National de Recherche en Génomique Humaine* (CNRGH, Evry, France). The DNA samples were plated onto 96-well or 48-well plates. In total, nine plates including 64 chips were used. These plates were analyzed in 4 batches. The ratios for sex (boy/girl) and recruitment centre (Poitiers/Nancy) were balanced for each chip. Fifteen samples were measured in quadruplicates and one sample in duplicate across batches, sample plates and chips to detect technical issues such as batch effects. The Illumina's Infinium HumanMethylation450 BeadChip, representing over 485,000 individual CpG sites, was used to assess levels of methylation in placenta samples following the manufacturer's instructions (Illuminas, San Diego, CA, USA). Raw signals of 450K BeadChips were extracted using the GenomeStudio® software (v2011.1. Illumina). The DNAm level of each CpG was calculated as the ratio of the intensity of fluorescent signals of the methylated alleles over the sum of methylated and unmethylated alleles (β value). All samples passed initial quality control and had on average>98% of valid data points (detection p-value < 0.01).

Genome-wide differential DNAm analyses

Genome-wide differential DNAm analyses were performed using robust linear regression models, adjusting for the main covariates. Maternal age (years), parity (dichotomous), maternal education (two-level factor) and maternal smoking during pregnancy were obtained by questionnaires. In addition to the indicated covariates, models were also adjusted for center of recruitment. We used principal components analysis to assess batch-related variation and corrected for batch effects using comBat. Six components capturing cellular heterogeneity were considered for adjusting. Most participants were of white-European ethnic origin.

Acknowledgements

We thank the families that participated in the study for their generous contribution. We thank the midwife research assistants (L. Douhaud. S. Bedel. B. Lortholary. S. Gabriel. M. Rogeon. and M. Malinbaum) for data collection and P. Lavoine for checking, coding, and entering data. The EDEN

mother-child cohort study group includes: I Annesi-Maesano, JY Bernard, J Botton, M-A Charles, P Dargent-Molina, B de Lauzon-Guillain, P Ducimetière, M de Agostini, B Foliguet, A Forhan, X Fritel, A Germa, V Goua, R Hankard, B Heude, M Kaminski, B Larroque, N Lelong, J Lepeule, G Magnin, L Marchand, C Nabet, F Pierre, R Slama, MJ Saurel-Cubizolles, M Schweitzer, O Thiebaugeorges.

Funding

This work was supported by the *Fondation de France* (n° 2012-00031593 and 2012-00031617), the French National Cancer Institute (INCa), the French Agency for National Research (ANR) (ANR-18-CE36-0005), the *Fonds de Recherche en Santé Respiratoire*, and the National Agency for Research (ANR-13-CESA-0011-02). The EDEN cohort has been funded by the Foundation for Medical Research (FRM), National Agency for Research (ANR), National Institute for Research in Public Health (IRESP: TGIR cohorte santé 2008 program), French Ministry of Health (DGS), French Ministry of Research, Inserm Bone and Joint Diseases National Research (PRO-A) and Human Nutrition National Research Programs, Paris–Sud University, Nestlé, French National Institute for Population Health Surveillance (InVS), French National Institute for Health Education (INPES), the European Union FP7 programs (FP7/2007-2013, HELIX, ESCAPE, ENRIECO, Medall projects), Diabetes National Research Program (through a collaboration with the French Association of Diabetic Patients (AFD)), French Agency for Environmental Health Safety (now ANSES), Mutuelle Générale de l'Education Nationale (MGEN), French National Agency for Food Security, and the French-speaking association for the study of diabetes and metabolism (ALFEDIAM). Funders had no influence of any kind on analyses or interpretation of results.

Gen3G (Genetics of Glucose regulation in Gestation and Growth)

Design and study population

The Genetics of Glucose regulation in Gestation and Growth (Gen3G) is a prospective observational cohort study aiming to increase our understanding of biological, environmental, and genetic determinants of glucose regulation during pregnancy and their impact on foetal development and was described in details previously[6]. In brief, we recruited a total of 1024 pregnant women aged ≥18 years old between January 2010 and June 2013 representing the general population of women in reproductive age receiving care at our institution. We excluded women if they had non-singleton pregnancy, known pre-pregnancy diabetes or overt diabetes diagnosed based on biochemical screening that we performed at first trimester. The study protocol was approved by the *Centre Hospitalier Universitaire de Sherbrooke* (CHUS) ethic committee board and every participant gave written informed consent before enrollment in the study, in accordance with the Declaration of Helsinki.

Placenta collection and DNA extraction

Trained research personnel collected fetal placenta tissue samples immediately after delivery (<30 min postpartum). A 1 cm^3 placenta tissue sample was collected approximately 5 cm from the umbilical cord insertion, from the fetal side of the placenta for each delivery. Placenta samples were collected by trained study staff and stored in RNAlater (Qiagen) at -80°C until DNA or RNA extraction occurred. We purified DNA and RNA from the placenta samples using the AllPrep DNA/RNA/Protein Mini Kit (Qiagen). Purity of extracted DNA was evaluated using a Spectrophotometer (Ultrospec 2000 UV/Visible; Pharmacia Biotech) with an absorbance ratio set at 260–280 nm as recommended.

Placental genome-wide DNAm data acquisition, quality control and normalization

Epigenome-wide DNA methylation measurements were performed on DNA from placenta samples using bisulfite conversion followed by quantification using the Infinium MethylationEPIC BeadChip (Illumina, San Diego, CA) that measures over 850,000 CpG sites at a single nucleotide resolution. Samples were randomly allocated to different plates and chips to minimize confounding. Methylation

data were imported into R for preprocessing using minfi. We performed quality control at the sample level, excluding samples that failed (n = 5), mismatch on genotype (n = 6) or sex (n = 1). A total of 448 individual women with high-quality DNA methylation data from placenta samples were retained for subsequent analyses.

Genome-wide differential DNAm analyses

We conducted Epigenome-Wide-Association Analyses (EWAS) of maternal body mass index (BMI) by fitting robust linear regression models using MASS package in R for each CpG on the normalized β-value scale adjusting for maternal age, parity, and smoking. Staff measured weight using a calibrated electronic scale and height using a wall stadiometer, and BMI was calculated as weight divided by squared height (kg/m^2) during the first visit, which occurred at a mean (range) gestational age of 9.9 (4.1-16.4) weeks. Maternal age was collected by questionnaire at the same first trimester visit. Parity was defined as the number of term pregnancies. Women reported smoking habits at first trimester and we classified them as "smoking" (current) or "non-smoking" (never or stopped before the visit). We did not adjust for maternal education since data was not collected during pregnancy. All included participants were of European origin. In the second model, we further adjusted for putative cellular heterogeneity using the reference-free cell mixture decomposition method, and we used three surrogate cell types.

Acknowledgements

Gen3G investigators acknowledge the Blood sampling in pregnancy clinic at the CHUS, and the assistance of clinical research nurses for recruiting women and obtaining consent for the study at the Research Center of CHUS. They also thank the CHUS Research in obstetrics services for organization of biosamples collection at delivery.

Funding

Gen3G was supported by F*onds de la recherche du Québec en santé* (FRSQ) operation grant #20697 (M.F.H); Canadian Institute of Health Research (CIHR) grant #MOP 115071 (M.F.H), and Diabète Québec grants (P.P. and L.B.). M.F.H. was supported by an American Diabetes Association (ADA) *Pathways to Stop Diabetes* Award (1-15-ACE-26).

GENEIDA (Genetics, Early Life Environmental Exposures and Infant Development in Andalusia)

Design and study population

The GENEIDA (Genetics, Early Life Environmental Exposures and Infant Development in Andalucía) project is a population-based prospective birth cohort of 802 mother-child pairs started in 2014 in the *El Poniente* (province of Almeria, South-Eastern Spain)[7]. This region is one of the most important intensive agricultural area in the country because of the large surface of plastic greenhouses. The aim of this project is to evaluate the role of early environmental exposures, such as chemical exposures, maternal stress, nutrition, on fetal and child growth and development. We are also studying the environmental influences on the placental epigenome and microbiome, and on maternal and children gut microbiome. The study was approved by the Research Ethics Committee of the province of Granada. The pregnant women received information of the study both written and orally, and they signed an informed consent.

Placenta collection and DNA extraction

Overall, 800 mother-child pairs were followed from until birth, and a total of 631 placentas were collected. For each placenta, a 1 cm^3 biopsy was taken in 4 well-defined regions of chorionic villus at the fetal side and ~5cm far from the umbilical cord insertion. Biopsies were collected during the first two hours after birth. Samples were washed with cold 1X PBS in order to avoid maternal contamination. They were stocked in RNAlater at 4°C during 4 weeks at most. After that, RNAlater was removed

and the samples were pulverized with liquid nitrogen and stocked in a biobank at -80°C until their processing. DNA extraction procedures were carried out in 25 mg of placental tissue. Placental tissues were homogenizated 5 min to 50 Hz with stainless steel beads and 1mL of slagboom buffer using the TissueLyser LT (Qiagen). After the tissues were broken up, they were incubated with 20 uL of Proteinase K (20 mg/mL) at 37°C overnight, followed by the digestion protocol proposed by Freeman et al. (2003)[8] and modified by Gómez-Martín et al. (2015)[9]. DNA quality was evaluated on a NanoDrop 2000 spectrophotometer (Thermo Fisher Scientific, Waltham, MA). Isolated genomic DNA was stored at -80ºC until further processing.

Placental genome-wide DNAmdata acquisition, quality control and normalization
Genome-wide DNAmanalysis was performed in 118 out of the 631 placental samples using the Infinium Human-Methylation450 Beadchip Array(Illumina, San Diego, CA USA) following manufacturer's recommendations. All samples were randomly loaded onto the arrays and processed by the same technician at the same time to minimize batch effects and processed blind to sample identification at the GENYO, Centre for Genomics and Oncological Research: Pfizer/ University of Granada/ Andalusian Regional Government (Spain). DNA (500 ng) was bisulfite-converted with EZ DNA Methylation Kit (Zymo Research, Orange, CA) according to Illumina's protocol for methylation profiling. BeadChips were scanned with an Illumina iScan software. Out of the 118 samples, 104 were of European origin. One sample was excluded because information on the maternal BMI was not available.

Genome-wide differential DNAm analyses
Raw IDAT files were processed to extract methylation levels (beta-values) for each sample and normalized using Functional Normalization[10]. Genome-wide differential DNAm analyses were performed using robust linear regression models, adjusting for main covariates of maternal age, parity, maternal education and maternal smoking during pregnancy, which were obtained by questionnaire administered to the mothers at first trimester of pregnancy. In addition, three components capturing cellular heterogeneity were considered for adjusting the model. All participants were of white-European ethnic origin.

Acknowledgements
The GENEIDA researchers would like to thank all the mothers who have participated in this study, without whom this study would not have been possible. We would like also show our gratitude to medical and fieldwork staff of "*Poniente Hospital*" for their assistance in contacting the families, administering the questionnaires and collecting biological samples. Finally, we also thank lab staff from GENYO for their assistance in processing placental samples.

Funding
GENEIDA Project was funded by grants from Spanish Ministry of Health (FIS-PI13/01559) and Andalusian Ministry of Health (PI-0405-2014; PS-0205-2016;PS-0508-2016).

Harvard Epigenetic Birth Cohort (HEBC)
Design and study population
Our study population consisted of women enrolled in the Harvard Epigenetic Birth Cohort (HEBC) at the Brigham and Women's Hospital in Boston, MA. The HEBC was initiated to study prenatal determinants of epigenetic marks in cord blood and placenta. Data and biospecimens for the HEBC were collected from June 2007 to June 2009 and include 1941 mother-child dyads. Details of the study protocol have been described previously[11]. The study was approved by the Institutional Review Board of the

Brigham and Women's Hospital and informed consent was obtained from all the participants included in this study.

Placenta collection and DNA extraction

Placenta samples were collected immediately after delivery. Placenta tissue was sampled by a 2-cm incision in the amnion. For this study, we analyzed samples (1 cm^3) taken from the fetal side near the umbilical cord. Genomic DNA were extracted from fresh frozen placenta tissue using the QIAamp DNA Mini Kit, following the manufacturer's instructions (Qiagen, Valencia, CA, USA).

Placental genome-wide DNAm data acquisition, quality control and normalization

Genome-wide DNA methylation was assayed for 263 participants by running 1 μg of placental DNA on the Illumina 450K Infinium Methylation BeadChip at the USC Epigenome Center (Los Angeles, CA, USA). Genomic DNA was bisulfite treated using the Zymo EZ-96 DNA methylation kit (Zymo Research, Irvine, CA, USA). The distribution of samples across chips was blinded by randomly sorting de-identified matched samples to reduce the likelihood of systematic technical bias. Out of the 263 samples, 19 were excluded from analysis for which more than 1% of CpG loci that did not meet our detection p-value cutoff (alpha-level=0.01), and 6 were excluded due to sex discordances. For the remaining subset, 25,326 probes were excluded based poor detection in at least one sample, resulting in an analytic set of 460,186 CpG loci.

Genome-wide differential DNAm analyses

Samples included in this analysis were combined from two batches; this potential batch effect was controlled for using ComBat. Our analysis was restricted to self-reported non-Hispanic white mothers who did not smoke during pregnancy (n=186). Cell composition was estimated using the reference-free method; six cell types were estimated in our dataset. Robust linear regression models were used for genome-wide DNA methylation analyses, adjusting for maternal age (continuous), and parity (none, one or more). Sensitivity analyses further adjusted for estimated cell composition.

Acknowledgements

We would like to thank the participants of the Harvard Epigenetics Birth Cohort and all staff who helped collect, store, and process the samples at the Brigham and Women's Hospital.

Funding

The Epigenetic Birth Cohort was funded in part by research grant R21CA128382 from the National Cancer Institute, National Institutes of Health.

INMA (Environment and Childhood Project)

Design and study population

The *Infancia y Medio Ambiente* (INMA) (Environment and Childhood Project) is a population-based mother-child cohort study in Spain that aims to study the role of environmental pollutants in air, water and diet during pregnancy and early childhood in relation to child growth and development.[12] More information about INMA project is available through our webpage http://www.proyectoinma.org/. The present study is based on the four *de novo* cohorts sited in Asturias, Gipuzkoa, Sabadell and Valencia and recruited between 2003 and 2008. The study was approved by the ethical committees of the centers involved in the study, and written informed consent was obtained from all the participants.

Placenta collection and DNA extraction

Overall, 2506 mother-child pairs were followed from until birth and a random selection of 489 placentas were collected. Collected placentas at the four areas of study were stored at -80ºC in a central

biobank until processing. Biopsies of approximately 5 cm^3 were obtained from the inner region of the placenta, approximately 1.0-1.5 cm below the fetal membranes, corresponding to the villous parenchyma, and at a distance of ~5 cm from site of cord insertion. 25 mg of placental tissue were used for DNA extraction, previously rinsed twice during 5 minutes in 0.8mL of 0.5X PBS in order to remove traces of maternal blood. Genomic DNA from placenta was isolated using the DNAeasy® Blood and Tissue Kit, (Qiagen, CA, USA). DNA quality was evaluated on a NanoDrop spectophotomer (Thermo Scientific, Waltham, MA, USA) and additionally 100 ng of DNA were run on 1.3% agarose gels to confirm that samples did not present visual signs of degradation. Isolated genomic DNA was stored at -20ºC until further processing.

Placental genome-wide DNAm data acquisition, quality control and normalization
Genome-wide DNAm examination was performed in 190 out of the 489 placentas samples using the Infinium Human-Methylation450 array (Illumina, San Diego, CA USA) following manufacturer's recommendations. All samples were randomly loaded onto the arrays and processed by the same technician at the same time to minimize batch effects and processed blind to sample identification at the University Medical Groningen Center UMCG Genome Analysis Facility (The Netherlands). DNA (500 ng) from each sample was treated by bisulfite conversion with the EZ-96 DNA Methylation Kit (Zymo Research) according to Illumina's protocol for methylation profiling. BeadChips were scanned with an Illumina iScan and image data was uploaded into the Methylation Module of Illumina's analysis software GenomeStudio (Illumina, San Diego, CA USA). Out of the 190 samples, 12 were excluded due to low performance (6.3%) and 10 due to sex discordances (5.3%).

Genome-wide differential DNAm analyses
Genome-wide differential DNAm analyses were performed using robust linear regression models, adjusting for main covariates. Maternal age, parity, maternal education and maternal smoking during pregnancy were obtained through a questionnaire administered to the mothers at first trimester. In addition to the indicated covariates, models were also adjusted for subcohort. The batch effect of 96-well bisulfite conversion plate (3 plates) was controlled using R ComBat Package (version 0.0.2). Two components capturing cellular heterogeneity were considered for adjusting. All participants were of white-European ethnic origin.

Acknowledgements
INMA researchers would like to thank all the participants for their generous collaboration. INMA researchers are grateful to Silvia Fochs, Nuria Pey, and Muriel Ferrer for their assistance in contacting the families and administering the questionnaires. The study was approved by the Ethical Committee of the Municipal Institute of Medical Investigation and by the Ethical Committee of the hospitals involved in the study. The pregnant women received information of the study both written and orally. Their informed consent of the participants was asked in each of the visits. A full roster of the INMA Project Investigators can be found at http://www.proyectoinma.org/presentacion-inma/listado-investigadores/en_listado-investigadores.html.

Funding
INMA was funded by grants from *Instituto de Salud Carlos III* (Red INMA G03/176, CB06/02/0041, PI18/01142, PI21/01491), Spanish Ministry of Health (FIS-PI041436, FIS-PI081151), *Generalitat de Catalunya*-CIRIT 1999SGR 00241, *Fundació La marató de TV3* (090430) and Department of Health of the Basque Government (GV-SAN2018111086, GV-SAN2019111085). We acknowledge the support from the Spanish Ministry of Science and Innovation and the State Research Agency through the "Centro de Excelencia Severo Ochoa 2019-2023" Program (CEX2018-000806-S), and the support from the Generalitat de Catalunya through the CERCA Program.

ITU (Intrauterine Sampling in Early Pregnancy Study)

Design and study population

The Intrauterine Sampling in Early Pregnancy Study is a prospective cohort study in Finland that aims to examine the biological maternal-placental-fetal mechanisms involved in the programming of health and disease after exposure to prenatal environmental adversity[13]. The sample comprises 944 women enrolled into the study in early pregnancy, their partners and children born alive between 2012-2017. The ITU research protocol has been approved by the Coordinating Ethics Committee of the Helsinki and Uusimaa Hospital District. Each ITU participant has signed a written informed consent form.

Placenta collection and DNA extraction

Of the 944 participants, placenta samples were available in 500. Within 0-2 hours of delivery, midwives took nine samples from the fetal-side of the placental tissue at 2-3 cm from umbilical cord insertion (3×3×3 mm each). These samples were stored in storage reagent meant to protect long-term RNA quality and integrity (Ciagen RNAprotect Tissue Reagent), first at +5°C, then at -20°C and -80°C for long-term storage. From the collected samples, DNA was extracted according to standard procedures. DNA was bisulfite-converted using the EZ-96 DNA Methylation kit (Zymo Research, Irvine, CA).

Placental genome-wide DNAm data acquisition, quality control and normalization

DNA (400ng) from placenta samples was used for bisulfite conversion using the EZ-96 DNA Methylation Kit (Zymo Research). The Infinium Methylation EPIC BeadChip Kit (Illumina Inc, San Diego, CA) was used to interrogate over 850,000 methylation sites quantitatively across the genome at single-nucleotide resolution. Preprocessing of methylation samples was conducted using the R package *minfi.*

Genome-wide differential DNAm analyses

Raw IDAT files were processed to extract methylation levels (beta-values) for each sample and normalized using Functional Normalization. Genome-wide differential DNAm analyses were performed using robust linear regression models, adjusting for main covariates and selection factor. Maternal age, parity and smoking during pregnancy were obtained from the Finnish Medical Birth Register and maternal education was self-reported in early pregnancy. The selection factor – chorosomal test in early pregnancy – was obtained from the recruitment files. We used principal components analysis to assess batch-related variation and corrected for batch effects using comBat. We adjusted for putative cellular heterogeneity using the reference-free cell mixture decomposition method. All participants were of white-European ethnic origin.

Acknowledgements

We would like to thank all of the midwives, obstetricians, and other medical staff, laboratory and research personnel, and administrative staff working in hospitals, antenatal and child wellbeing clinics, municipalities, national registers, and other institutions, whose efforts have made this research possible. We would also like to thank a number of research assistants who during the study period participated in data collection. Finally, and most importantly, we wish to express our deepest gratitude to all the mothers, partners and children who have participated in the ITU study.

Funding

The ITU study is funded by Academy of Finland (award numbers: 284859 and 312670).

NHBCS (The New Hampshire Birth Cohort Study)

Design and study population

The New Hampshire Birth Cohort Study (NHBCS) is an ongoing birth cohort that recruits pregnant women between the ages of 18 and 45 years, who are attending one of the study clinics in New Hampshire for prenatal care[14]. This study was initiated to examine the impacts of environmental toxicants on growth and development, and thus only included those mothers who used an unregulated well as the primary source of drinking water. All participants provided written informed consent in accordance with the requirements of the Institutional Review Board (IRB) of Dartmouth College. Placenta (n=343) were sampled for genetic and epigenetic assays between February 2012 and September 2013. Interviewer administered questionnaires and medical record abstraction were utilized to collect sociodemographic, lifestyle, and anthropometric data.

Placenta collection and DNA extraction
Placental tissues were biopsied from the fetal side adjacent to the cord insertion site after removing maternal *decidua* and was performed within 2 hours of delivery. Samples were placed in RNAlater (Life Tecnologies, Carlsbad, CA) then frozen at -80°C. Both DNA was extracted (Norgen Biotek, Thorold, ON) and quantified via the Qubit Flourometer (Life Technologies), then subsequently stored at -80°C.

Placental genome-wide DNAm data acquisition, quality control and normalization
We measured DNAm via Illumina Infinium HumanMethylation450K BeadArray (Illumina, San Diego, CA) at the University of Minnesota Genomics Center. The EZ Methylation kit (Zymo Research, Irvine, CA) was utilized for bisulfite modification, samples were randomized across multiple batches, while batch variables were recorded. Data were assembled using BeadStudio (Illumina). The raw array data have been deposited at the NCBI Gene Expression Omnibus (GEO) for NHBCS via accession number GSE71678.

Genome-wide differential DNAm analyses
We used principal components analysis to assess batch-related variation and corrected for batch effects using ComBat. Genome-wide differential DNAmanalyses were performed using robust linear regression models, adjusting for maternal age (years), parity (dichotomous), maternal education (three-level factor) and maternal smoking during pregnancy, which were measured via questionnaire. Two components capturing cellular heterogeneity were considered for adjustment. All participants were of white-European ethnic origin.

Funding
This work was supported by the National Institutes of Health [NIH-NIGMS P20 GM104416, P01 ES022832] and by the United States Environmental Protection Agency [US EPA grant RD83544201]. Its contents are solely the responsibility of the grantee and do not necessarily represent the official views of the US EPA. Further, the US EPA does not endorse the purchase of any commercial products or services mentioned in the presentation.

RICHS (Rhode Island Child Health Study)
Design and study population
The Rhode Island Child Health Study (RICHS) is a study of mother-infant pairs with non-pathological pregnancies that were enrolled from the Women and Infants' Hospital in Providence, RI, USA between September 2010 and February 2013[15]. Mothers younger than 18 years of age, with life threatening conditions, pregnancies resulting in preterm birth (< 37 weeks gestation), or congenital/chromosomal abnormalities were excluded. All protocols were approved by the institutional review boards (IRB) at the Women and Infants Hospital of Rhode Island and Emory University and all participants provided written informed consent. The study enrolled term infants born small for gestational age (SGA < 10th birthweight percentile), large for gestational age (LGA > 90th

birthweight percentile), and infants adequate for gestational age matched on sex, gestational age, and maternal age. Sociodemographic and lifestyle data were collected via questionnaire while anthropometric and medical history data were obtained via structured medical record abstraction. Due to the vast majority of participants from other cohorts consisting of participants of white European ancestry, we restricted our analysis to those mothers that self-reported as white.

Placenta collection and DNA extraction

Placental tissues were biopsied from the fetal side adjacent to the cord insertion site after removing maternal *decidua* and was performed within 2 hours of delivery. Samples were placed in RNAlater (Life Technologies, Carlsbad, CA) then frozen at -80°C. DNA was extracted (Norgen Biotek, Thorold, ON) and quantified via the Qubit Flourometer (Life Technologies), then subsequently stored at -80°C.

Placental genome-wide DNAm data acquisition, quality control and normalization.

We measured DNAm via Illumina Infinium HumanMethylation450K BeadArray (Illumina, San Diego, CA) at the University of Minnesota Genomics Center. The EZ Methylation kit (Zymo Research, Irvine, CA) was used for bisulfite modification, samples were randomized across multiple batches, batch variables were recorded to allow for corrections. Data were assembled using BeadStudio (Illumina).
Array image files (IDAT) preprocessing and data QC was performed in R using the minfi package (PMID: 24478339) included the following steps: background correction, functional normalization (PMID: 25599564), detection and exclusion of poor-quality probes (p-value < 0.01), assessment of concordance between array-inferred sex and phenotypic sex, probe type dye bias adjustment with BMIQ (PMID: 23175756) and batch effects adjustment using ComBAT (PMID: 16632515). Before association analyses we removed the following probes: X or Y linked, probed with SNPs, and known to be cross hybridizing (PMID:23314698). Raw array data are available via the NCBI Gene Expression Omnibus (GEO) for RICHS (GSE75248).

Genome-wide differential DNAm analyses

Genome-wide differential DNAm analyses were performed using robust linear regression models, adjusting for maternal age (years), parity (dichotomous), maternal education (three-level factor) and maternal smoking during pregnancy, which were measured via questionnaire. Two components capturing cellular heterogeneity were considered for adjusting.

Funding

The RICHS study is supported by the National Institutes of Health (NIH) with the following grants: NIH-NIMH R01MH094609, NIH-NIEHS R01ES022223, and NIH-NIEHS R24ES028507. CL is funded through NIH-NICHD R00HD097286.

Supplementary Figure 1: Forest plots of the leave-one-out analysis showing the fixed effects meta-analysis estimates of association between maternal ppBMI and placental DNAm, including data from the MARBLES cohort, showing the strong deviation of this dataset, so that it was removed from the final study.

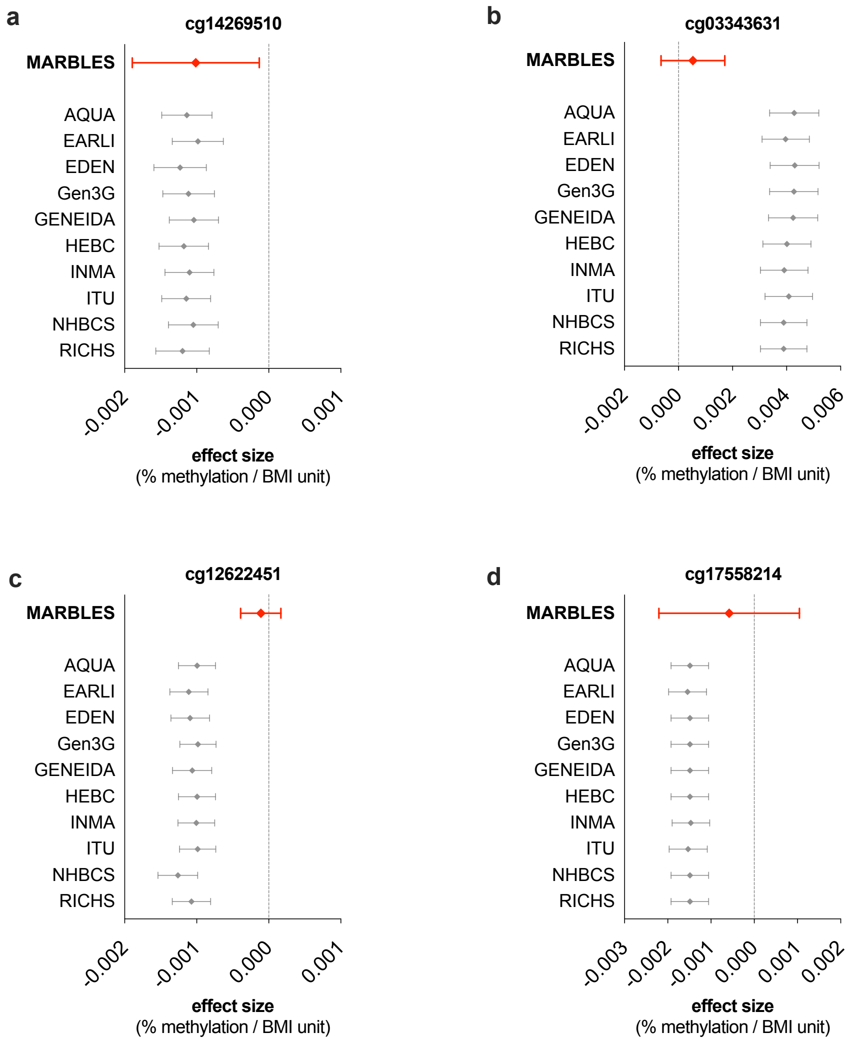

Association of **a** cg14269510, **b** cg03343631, **c** cg12622451, **d** cg17558214 with maternal ppBMI. In all panels, cohort names indicate the cohort excluded in each row, and error bars represent the 95% confidence interval of the effect size.

Supplementary Figure 2: Correlation analysis of the effect sizes of the differentially methylated hits in the models adjusted and not adjusted by potential cellular heterogeneity.

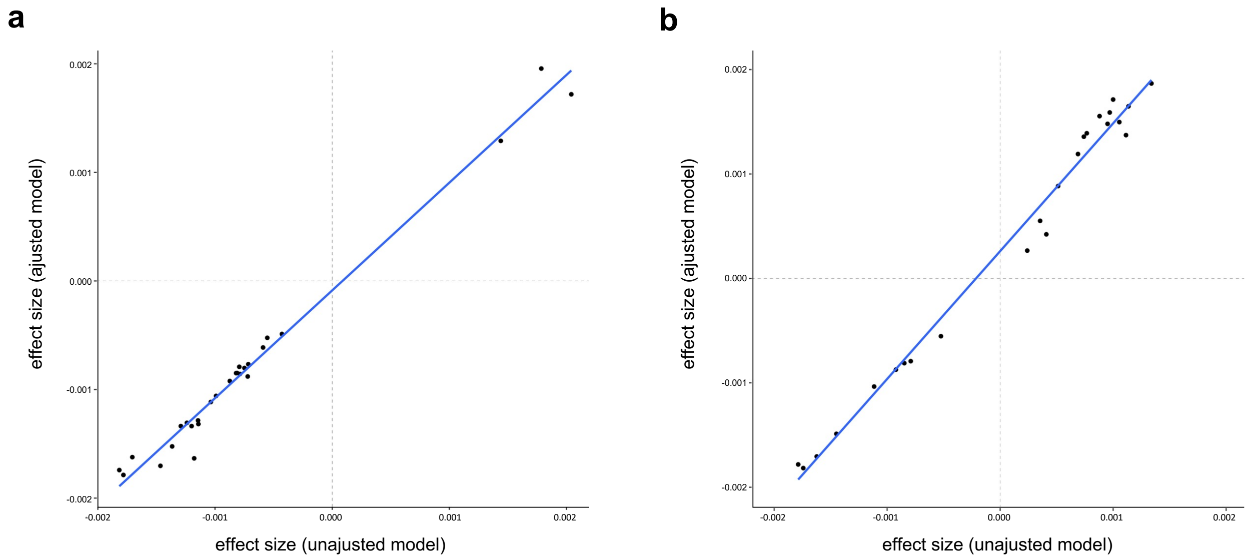

Correlation between the effect sizes of the significant hits (Bonferroni corrected, nominal p value < 1.2e -7) identified in models **a** adjusted and **b** unadjusted for cellular heterogeneity, and the effect sizes of the same CpGs in the other model, respectively. In both panels, the black points represent the observed data and the predicted regression line is shown in blue.

Supplementary Figure 3: Forest plots of the leave-one-out analysis showing the fixed effects meta-analysis estimates of association between maternal ppBMI and placental DNAm.

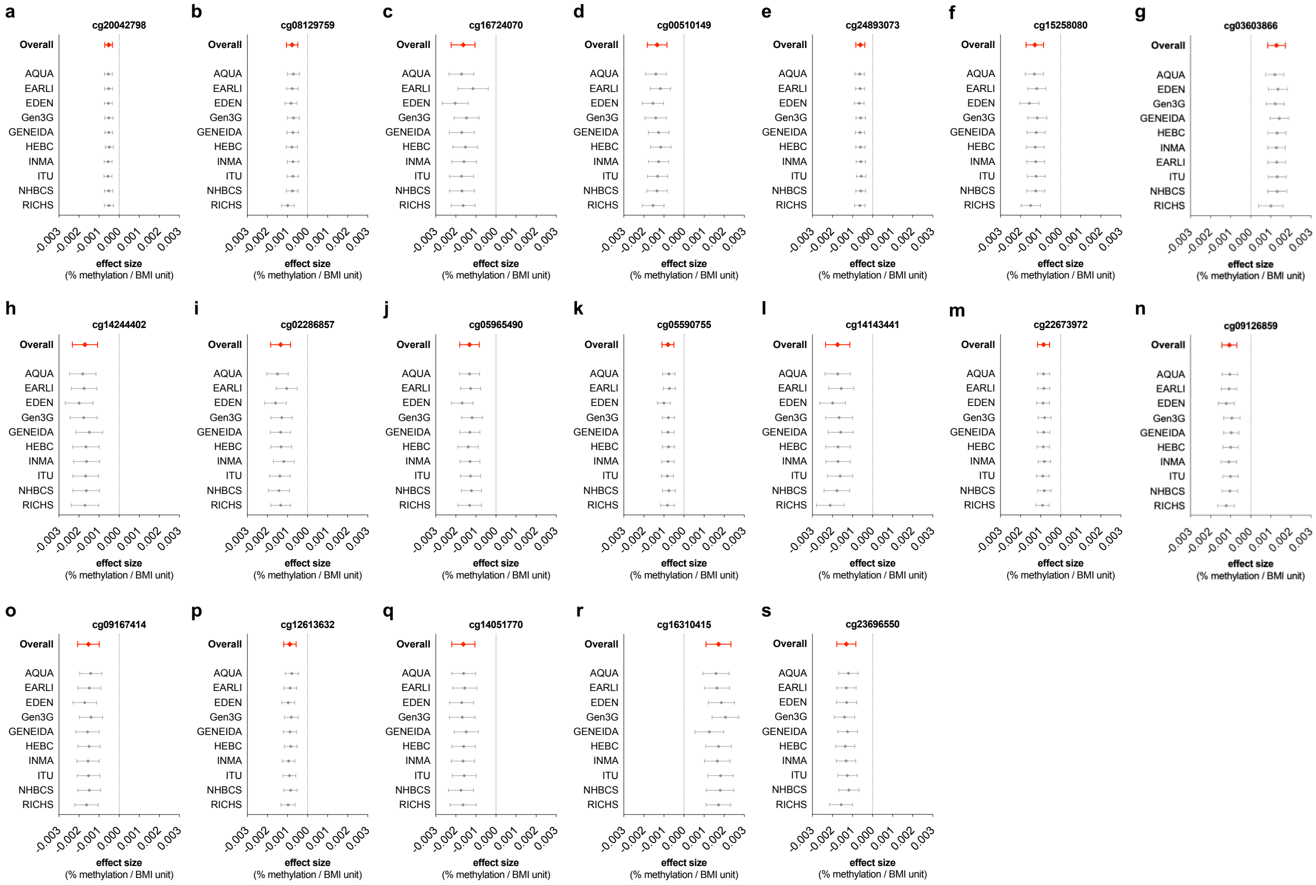

Association of **a** cg20042798, **b** cg08129759, **c** cg16724070, **d** cg00510149, **e** cg24893073, **f** cg14244402, **g** cg02286857, **h** cg05965490, **i** cg05590755, **j** cg15258080, **k** cg03603866, **l** cg14143441, **m** cg22673972, **n** cg09126859, **o** cg09167414, **p** cg12613632, **q** cg14051770, **r** cg16310415 and **s** cg23696550 with maternal ppBMI. In all panels, cohort names indicate the cohort excluded in each row, and error bars represent the 95% confidence interval of the effect size.

Supplementary Figure 4: Proportion of CpGs annotated to islands and open sea regions in the whole array probe set and among maternal ppBMI associated hits.

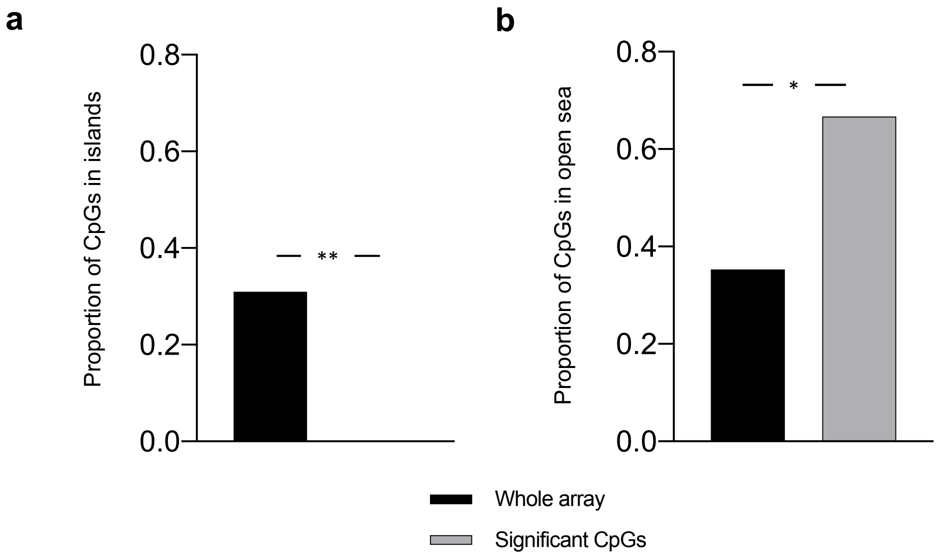

Maternal ppBMI-associated CpGs were **a** depleted in CpG islands (**χ^2 p-value = 8.4e-04) and **b** enriched for open sea regions (*χ^2 p-value = 1.3e-03).

Supplementary References

1. Muggli, E. *et al.* Study protocol: Asking QUestions about Alcohol in pregnancy (AQUA): a longitudinal cohort study of fetal effects of low to moderate alcohol exposure. *BMC Pregnancy Childbirth* **14**, 302 (2014).

2. Ollikainen, M. *et al.* DNA methylation analysis of multiple tissues from newborn twins reveals both genetic and intrauterine components to variation in the human neonatal epigenome. *Human Molecular genetics* **19**, 4176-4188 (2010).

3. Newschaffer, C.J. *et al*. Infant siblings and the investigation of autism risk factors. *J. Neurodev. Disord.* **4**, 7 (2012).

4. Heude, B. *et al.* Cohort Profile: The EDEN mother-child cohort on the prenatal and early postnatal determinants of child health and development. *International journal of epidemiology* **45**, 353-363 (2016).

5 Abraham, E. *et al*. Pregnancy exposure to atmospheric pollution and meteorological conditions and placental DNA methylation. *Environment international* **118**, 334-347 (2018).

6. Guillemette, L. *et al*. Genetics of Glucose regulation in Gestation and Growth (Gen3G): a prospective prebirth cohort of mother-child pairs in Sherbrooke, Canada. *BMJ open* **6**, e010031 (2016).

7. Aguilar-Lacasaña, S. *et al.* Methylenetetrahydrofolate Reductase (MTHFR) Gene Polymorphism and Infant's Anthropometry at Birth. *Nutrients* **13**, 831. doi:10.3390/nu13030831 (2021).

8. Freeman, B. *et al*. DNA from buccal swabs recruited by mail: evaluation of storage effects on long-term stability and suitability for multiplex polymerase chain reaction genotyping. *Behav. Genet.* **33**, 67-72 (2003).

9. Gomez-Martin, A. *et al*. Polymorphisms of pesticide-metabolizing genes in children living in intensive farming communities. *Chemosphere* **139**, 534-540 (2015).

10. Fortin, J. P. *et al.* Functional normalization of 450k methylation array data improves replication in large cancer studies. *Genome biology* **15**, 503 (2014).

11. Michels, K. B., Harris, H. R. & Barault, L. Birthweight, maternal weight trajectories and global DNA methylation of LINE-1 repetitive elements. *PLoS One* **6,** e25254 (2011).

12. Guxens, M. *et al.* Cohort Profile: the INMA--INfancia y Medio Ambiente--(Environment and Childhood) Project. *International journal of epidemiology* **41**, 930-940 (2012).

13. Dieckmann, L. *et al*. Characteristics of epigenetic aging across gestational and perinatal tissues. *Clin. Epigenetics* **13**, 97 (2021).

14. Gilbert-diamond, D., Emond, J. A., Baker, E. R., Korrick, S. A. & Karagas, M. R. Relation between in Utero Arsenic Exposure and Birth Outcomes in a Cohort of Mothers and Their Newborns from New Hampshire. *Environ. Health Perspect.* **124**, 1299–1307 (2016).

15. Appleton, A. A. *et al.* Prenatal Programming of Infant Neurobehaviour in a Healthy Population. *Paediatr. Perinat. Epidemiol.* **30**, 367–375 (2016).
