# Supplementary material for: A meta-analysis of pre-pregnancy maternal body mass index and placental DNA methylation identifies 27 CpG sites with implications for mother-child health
Source: Commun Biol. 2022 Nov 30;5:1313. doi: 10.1038/s42003-022-04267-y (PMC9709064; doi:10.1038/s42003-022-04267-y)
Supplement: Supplementary file 3 — Description of Additional Supplementary Files [file 42003_2022_4267_MOESM3_ESM.pdf]

Description of Additional Supplementary Files

File name: Supplementary Data 1
Description: Distribution of maternal BMI, demographic variables, birth outcomes, and covariates, by cohort. IQR = Inter Quartile Range.

File name: Supplementary Data 2
Description: Effective number of probes, number of Bonferroni significant CpGs and lambda values for the cohort-specific models, adjusted and unadjusted for cellular heterogeneity. The number of components capturing cellular heterogeneity used for each cohort is also defined.

File name: Supplementary Data 3
Description: Meta-analysis results for the association between maternal ppBMI and placental DNAm, adjusted for cellular heterogeneity.

File name: Supplementary Data 4
Description: Meta-analysis results for the association between maternal ppBMI and placental DNAm, unadjusted for cellular heterogeneity.

File name: Supplementary Data 5
Description: Enrichment for functional pathways among CpGs associated with maternal ppBMI, annotated to the closest gene from the Illumina annotation file.

File name: Supplementary Data 6
Description: SNPs associated with birth weight and their proximity to CpGs associated with maternal ppBMI in placenta.

File name: Supplementary Data 7
Description: CpGs associated with maternal ppBMI in placenta and cord blood.

File name: Supplementary Data 8
Description: CpG positions discarded due to a detection p-value > 0.01 in each cohort.

File name: Supplementary Data 9
Description: Numerical source data for Figure 3.
