# Supplementary material for: A meta-analysis of pre-pregnancy maternal body mass index and placental DNA methylation identifies 27 CpG sites with implications for mother-child health
Source: Commun Biol. 2022 Nov 30;5:1313. doi: 10.1038/s42003-022-04267-y (PMC9709064; doi:10.1038/s42003-022-04267-y)
Supplement: Supplementary file 13 — Reporting Summary [file 42003_2022_4267_MOESM13_ESM.pdf]

nature portfolio

Corresponding author(s): Jose Ramon Bilbao
Marie France Hivert

Last updated by author(s): 11/13/2022

Reporting Summary

Nature Portfolio wishes to improve the reproducibility of the work that we publish. This form provides structure for consistency and transparency in reporting. For further information on Nature Portfolio policies, see our Editorial Policies and the Editorial Policy Checklist.

Statistics

For all statistical analyses, confirm that the following items are present in the figure legend, table legend, main text, or Methods section.

n/a	Confirmed	
☐	☒	The exact sample size (n) for each experimental group/condition, given as a discrete number and unit of measurement
☐	☒	A statement on whether measurements were taken from distinct samples or whether the same sample was measured repeatedly
☐	☒	The statistical test(s) used AND whether they are one- or two-sided<br>*Only common tests should be described solely by name; describe more complex techniques in the Methods section.*
☐	☒	A description of all covariates tested
☐	☒	A description of any assumptions or corrections, such as tests of normality and adjustment for multiple comparisons
☐	☒	A full description of the statistical parameters including central tendency (e.g. means) or other basic estimates (e.g. regression coefficient) AND variation (e.g. standard deviation) or associated estimates of uncertainty (e.g. confidence intervals)
☐	☒	For null hypothesis testing, the test statistic (e.g. F, t, r) with confidence intervals, effect sizes, degrees of freedom and P value noted<br>*Give P values as exact values whenever suitable.*
☒	☐	For Bayesian analysis, information on the choice of priors and Markov chain Monte Carlo settings
☒	☐	For hierarchical and complex designs, identification of the appropriate level for tests and full reporting of outcomes
☐	☒	Estimates of effect sizes (e.g. Cohen's d, Pearson's r), indicating how they were calculated

Our web collection on statistics for biologists contains articles on many of the points above.

Software and code

Policy information about availability of computer code

Data collection	All software packages and programs used for data collection are freely available, and can be found via the citations in the manuscript.
Data analysis	All software packages and programs used in the analyses are freely available, and can be found via the citations in the manuscript. The specific code generated for those analyses is available upon request to the corresponding authors.

For manuscripts utilizing custom algorithms or software that are central to the research but not yet described in published literature, software must be made available to editors and reviewers. We strongly encourage code deposition in a community repository (e.g. GitHub). See the Nature Portfolio guidelines for submitting code & software for further information.

Data

Policy information about availability of data

All manuscripts must include a data availability statement. This statement should provide the following information, where applicable:

- Accession codes, unique identifiers, or web links for publicly available datasets
- A description of any restrictions on data availability
- For clinical datasets or third party data, please ensure that the statement adheres to our policy

Cohort-specific summary statistics will be made publicly available upon acceptance.

Field-specific reporting

Please select the one below that is the best fit for your research. If you are not sure, read the appropriate sections before making your selection.

☒ Life sciences ☐ Behavioural & social sciences ☐ Ecological, evolutionary & environmental sciences

For a reference copy of the document with all sections, see nature.com/documents/nr-reporting-summary-flat.pdf

Life sciences study design

All studies must disclose on these points even when the disclosure is negative.

Sample size	No sample-size calculation was performed. Cohorts that are members of the PACE consortium that had existing DNAm data from placental tissue obtained with the Illumina 450K or EPIC BeadChips, and had maternal BMI information prior to or at the beginning of pregnancy were invited to participate, and samples meeting those criteria were included.
Data exclusions	One of the participating cohorts: Markers of Autism Risk in Babies-Learning Early Signs (MARBLES) was excluded from the final analyses because of its limited sample size and results were inconsistent with other cohorts.
Replication	Each of the participant cohorts performed technical replications of their genomic experiments as detailed in the "Supplementary Methods" section.
Randomization	This is an observational study, and no allocation into experimental groups was performed. Results from each of the cohorts were meta analyzed, and cohort- and population-related covariates were accounted for. Potential batch effects due to
Blinding	Blinding is not applicable, since this is a population-based study

Reporting for specific materials, systems and methods

We require information from authors about some types of materials, experimental systems and methods used in many studies. Here, indicate whether each material, system or method listed is relevant to your study. If you are not sure if a list item applies to your research, read the appropriate section before selecting a response.

Materials & experimental systems

n/a	Involved in the study	
☒	☐	Antibodies
☒	☐	Eukaryotic cell lines
☒	☐	Palaeontology and archaeology
☒	☐	Animals and other organisms
☐	☒	Human research participants
☒	☐	Clinical data
☒	☐	Dual use research of concern

Methods

n/a	Involved in the study	
☒	☐	ChIP-seq
☒	☐	Flow cytometry
☒	☐	MRI-based neuroimaging

Human research participants

Policy information about studies involving human research participants

Population characteristics	Eleven North-American, Australian, and European studies (N=2,631) contributed to the epigenome-wide association study (EWAS) to determine the associations of maternal ppBMI on placental DNAm. Each cohort is described in the "Supplementary Methods" section.
Recruitment	Studies are population-based birth cohorts, and recruitment for each of them is described in the "Supplementary Methods" section, and in the references provided.
Ethics oversight	All cohorts obtained ethics approval and informed consent from participants prior to data collection through their Institutional Ethics Boards

Note that full information on the approval of the study protocol must also be provided in the manuscript.
